# Atlasprofilax: a new promising treatment for chronic cervicobrachialgia. A qualitative-quantitative research of a retrospective longitudinal section, with a cause-effect approach

**DOI:** 10.3389/fmedt.2025.1513155

**Published:** 2025-03-18

**Authors:** R. Rezende, J. G. León Higuera, L. Manent, K. Lewis, O. Angulo

**Affiliations:** ^1^ Clínica Ortopédica Especializada de Governador Valadares, Governador Valadares, Brazil; ^2^ Department of Sports Medicine and Rehabilitation, The San José Children’s University Hospital, Fundacion Universitaria de Ciencias de la Salud, Bogotá D.C., Colombia; ^3^ Independent Researcher, Los Angeles, CA, United States; ^4^ Universidad Cooperativa de Colombia, Sede Villavicencio, Colombia

**Keywords:** Atlasprofilax, cervicobrachialgia, neck pain, fascia, pain

## Abstract

**Objectives:**

To evaluate the effectiveness of the Atlasprofilax intervention in the treatment of chronic cervicobrachialgia in a cohort of 162 patients. The assessment focused on measuring pain reduction, overall patient satisfaction, and improvements in the range of motion of the neck and the affected upper-limb.

**Methods:**

A retrospective, open-label, qualitative-quantitative longitudinal cut study was conducted in an orthopedic medical center in Brazil from June 2016 to July 2017. A total of 162 Brazilian patients with diagnosed chronic cervicobrachialgia were treated with a single session of non-invasive device-mediated treatment (Atlasprofilax method) that utilizes mechanotransductive vibropercussion on the suboccipital myofascia for approximately eight minutes. Patient conditions were established at baseline, and three follow-up assessments were conducted at 1, 6, and 9 months after treatment to evaluate the endpoints. Primary endpoints included changes in the cervical VAS pain and brachial VAS pain, while secondary endpoints included changes in the range of motion of the neck and affected upper limb, as well as patient satisfaction. A single blinded examiner conducted the evaluations at baseline and follow-up assessments, and the intervention was performed by an orthopedic doctor specializing in shoulder surgery.

**Results:**

The primary endpoints showed a significant reduction in pain. The mean cervical VAS pain score at baseline was 7.15 ± 2.15 [median VAS 8 (6;8)], which reduced to 1.47 ± 1.04 [median 0.5 (0/2)] at month 9 [mean reduction −5.67 ± 2.30 and median −6 (−7/−4), *p* < 0.0001]. Fifty percent of the patients reported no pain on the VAS at the 9-month follow-up. The mean brachial VAS pain score at baseline was 6.16 ± 2.31 [median 6 (3;8)], which reduced to 0.33 ± 1.79 [median 0 (0;2)] at month 9 [mean reduction −5.83 ± 2.35; median reduction −6 (−8/−4), *p* < 0.0001]. At the 9-month follow-up, 88.89% of patients reported no brachial pain on the VAS. Secondary endpoints indicated a marked improvement in the average range of motion of the neck and upper limb in all subtypes of measurements. Additionally, 87.04% of patients reported satisfaction with the therapy and an improvement in their daily activities. No side-effects were observed.

**Conclusions:**

AtlasProfilax is nowadays a good option as an intervention when it comes to pain control and activities of daily living.

## Graphical Abstract

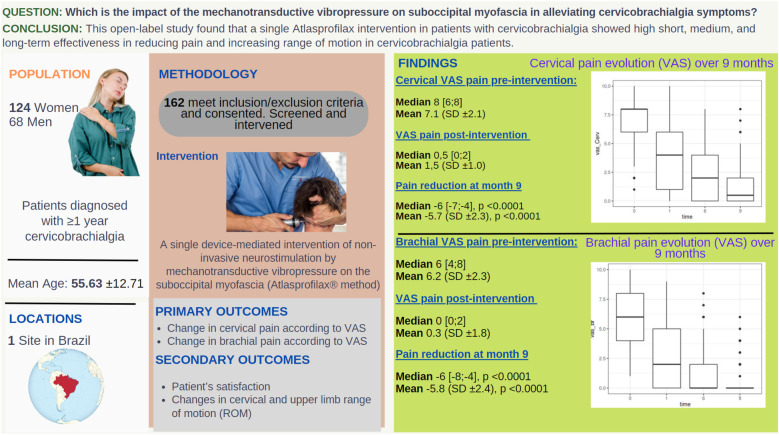

## Highlights

• Cervicobrachialgia is a common condition among the population, associated with pain and upper limb impairment, and can become chronic.
• Its therapeutic management is usually difficult and is mostly treated with pharmacological, surgical, and manual non-invasive physical therapy approaches.
• 162 patients suffering from chronic cervicobrachialgia were treated with a single, non-invasive, device-mediated technique that uses mechanotransductive vibropercussion on the suboccipital myofascia.
• Preliminary results indicate that this intervention (Atlasprofilax method) is highly effective and long-lasting in reducing pain, symptoms, and increasing the range of motion of the neck and upper limbs, resulting in high patient satisfaction.

## 1 Introduction

Cervicobrachialgia is characterized by cervical pain that radiates to the upper limb and is accompanied by paresthesia of one or more fingers (1). The brachial plexus is responsible for innervating the entire upper limb (2). Alterations in the fascial and muscle chains surrounding the brachial plexus can cause upper limb symptoms such as cervicobrachialgia. The interactions between the peripheral nervous system, fascia, and muscles are complex and essential for developing effective treatments for musculoskeletal and neurological conditions. When the myofascial or nervous system is injured, it can result in mechanical deformation of the nerve fibers, which reduces the axoplasmic flow (3), alters nervous function, and causes sensory and motor symptoms (4). Therefore, it is appropriate to consider the PNS, fascia, and muscles as part of a unique biomechanical continuum that interacts to produce movement and regulate physiological functions in the body. The PNS can be affected through the transmission of forces and movements generated by the connective envelopes present in the nerve cells (5, 6). In addition to its mechanical functions, fascia also plays a significant role in metabolic and biomechanical interactions with the extracellular matrix (7, 8), soft tissues, joints, and the body as a whole (9). Peripheral nervous system function is important in treating cervicobrachialgia. Neural mobilization techniques can restore the musculoskeletal structures involved in PNS biomechanics (10). However, evidence for the effectiveness of non-invasive management of this condition is inconclusive (11), and pharmacologic treatments have shown better effectiveness than neural mobilization (12). Common interventions do not seem to have a clear mid- or long-term impact. Novel non-invasive approaches are required for more effective treatment.

Atrophy of the suboccipital muscles have been linked with chronic neck pain (13, 14), which is commonly associated with brachial pain (11, 15). Abnormalities in the deep cervical fascia and its continuum have been associated to cervicobrachialgia (16). Mechanical forces can impact cell-cell junctions and cytoskeleton contractibility (17), influencing tissue-scale tension generation critical for homeostasis and morphogenesis. Fascia, which regulates contractile force and tissue stiffness mechanisms (18, 19), relies on specific mechanisms of mechanotransduction that respond to external stimuli, including sustained vibropressure, which can even affect gene expression.

The hypothesis that a somatic dysfunction at the craniocervical junction involving suboccipital myofascia atrophy and fascial continuum entrapment may be correlated with cervicobrachialgia symptoms cannot be dismissed. Therefore, the application of external vibropercussive and mechanotransductive stimuli on the suboccipital myofascia could potentially enhance self-regulation and restoring mechanisms of the soft tissue, resulting in an improvement of cervicobrachialgia symptoms.

The Atlasprofilax method is a treatment that has been shown to have a good safety profile and to provide benefits for cervicobrachialgia (20), as well as other spine-related disorders (21–23) and temporomandibular joint disorders (TMJD) (24). TMJDs are often linked to problems with the cervical spine (25), such as limited range of motion (26), neck disability index, muscle tenderness, and cervical curvature angle (27), as well as Atlas-Axis asymmetries (28). The Atlasprofilax method has also demonstrated improvements in Atlas-Axis asymmetries (20, 22), cervicobrachialgia (20), miofascial pain syndrome (23), and fibromyalgia syndrome (FMS) (29), a condition that is closely related to chronic cervical myofascial pain (30). As such, we aimed to investigate the impact of neural mobilization and myofascial release using vibropressure and mechanotransduction (Atlasprofilax method) on the suboccipital region, and its effectiveness in reducing cervical and brachial pain and improving mobility range in the neck and the affected upper limb in patients with cervicobrachialgia symptoms.

## 2 Methods

### 2.1 Ethics committee approval

Approved: No. BIO359.

The above, within the framework of resolution 8430 of October 4th, 1993 of the Ministry of Health of the Republic of Colombia, which establishes the scientific, technical and administrative standards for health research, according to Chapter 1 Art. 5–16 and in accordance with international requirements: Declarations of Helsinki, Finland, latest version year 2000, international ethical guidelines for biomedical research and experimentation in human beings, prepared by the Council of International Organizations of Medical Sciences (CIOMS) in collaboration with the World Health Organization (WHO), Geneva 1993 and 2002.

### 2.2 Study design

The study design was a qualitative-quantitative research of a retrospective longitudinal cut, with a cause-effect approach. The study sample consisted of 162 patients who suffered from cervicobrachialgia, including 124 women (75.54%) and 68 men (24.45%). The median age was 56 years (Q1–Q3; 47.25, 73), and the mean age was 55.63 years (SD 12.71 years) ranging from 19 to 88 years old. In the majority of cases (84.2%), the affected upper limb was the dominant limb. The study was conducted over a period of 9 months.

Degenerative processes such as disc osteophytes were found in 86 patients, while 9 patients had a cervical herniated disc, and 67 patients had bad posture, trauma, and other musculoskeletal disorders. Among the affected upper limbs, 41.98% were on the left side (*n* = 68/162). The median duration of symptoms linked to cervicobrachialgia was 4 years (Q1–Q3; 2–5), and the mean duration was 4.204 years (SD, 3.099 years), varying from 1 year to 20 years of evolution.

Patients were selected randomly based on specific criteria for this study with following inclusion criteria: patients diagnosed with cervicobrachialgia for at least a year, with cervical origin clinical signs such as muscle weakness, decreased reflexes, pain, and/or paresthesia at the end of the dermatome, and limited cervical spine and upper limb range of motion. Patients who did not receive any other type of treatment, including drug treatment, were included, regardless of race, gender, and availability. Exclusion criteria included recent cochlear or retinal implants, aggravation of the condition, pregnancy, and any potential contraindications to the intervention. Patients who planned to undergo other therapies during the 9-month study period were also excluded.

### 2.3 Methodology and measurements

**Figure 1:**
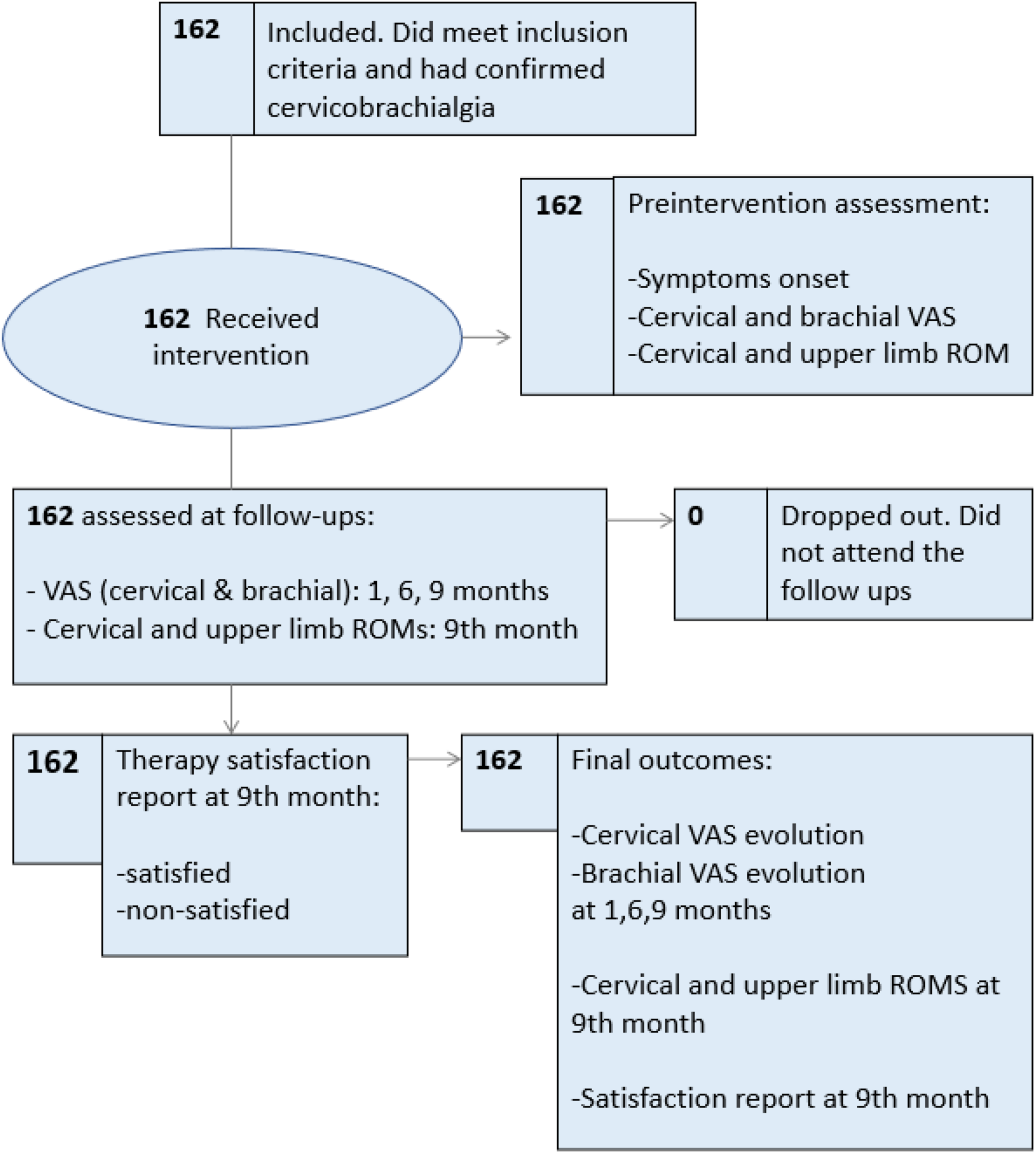
Study flowchart.

The study flowchart is depicted in Figure 1. The study collected personal data and evaluated patients' condition using the Visual Analog Scale (VAS) for pain intensity, goniometer for measuring cervical and upper limb range of motion, and specific orthopedic tests to confirm diagnosis such as the Valsalva Test, the Spurling Test, the Distraction Test, and the Maximum Foraminal and Compression Test, as described by Rambaut (31), were performed. Cervical and brachial pain using VAS was measured before and after intervention at month 1, 6, and 9. Range of motion was measured at baseline and 9th month follow-up. Patient satisfaction was assessed at the end of the 9-month study. Patients attended the same medical service between June 2016 and July 2017 and met the inclusion and exclusion criteria. All cases included in this work presented complaints of neck pain, sensory disturbances in the upper limb (pain/paresthesia) and some motor (weakness) and autonomic (vasomotor) disturbances. The Atlasprofilax treatment was performed only once during a single session right after baseline measurements, and the subsequent endpoints were gradually assessed and measured in all cases during follow-ups.

### 2.4 Intervention

The Atlasprofilax intervention uses a device-mediated with non-invasive vibropressure seeking a mechanotransductive effect on some structures and receptors of the suboccipital myofascia which can extend along the myofascial continuum (32). This means that, this tissue vibration would translate into a biomolecular and cellular effect, emphasizing in a restorative and regenetive effect that lasts for at least 12 months; furthermore, a pain lowering benefit. The treatment consists in a previous functional analysis that measures dysfunctional patterns in the fascial tissue especially in the upper neck region but also distally. Then, a special apparatus employing non-invasive digital or analogic controlled mechanotransductive vibropressure is used on specific spots of the suboccipital region. Atlasprofilax's treatment protocols are adapted following anthropometric criteria and depending on neck's morphology of the patient and its general health condition. Specific angles, sequences, time of pressure, specific-dosed amount of pressure, different rubber tips and different frequencies in Hz, are applied using this apparatus on precise key spots in the suboccipital area in order stimulate some muscle and fascia receptors and spindles during approximately 8 to 9 min.

The aim of this Method is to produce precise stimulus seeking to restore tissue mechanical abnormalities in the suboccipital myofascia region (32) and enhance cell metabolism in fascia and muscles and mobilizing neural tissue indirectly along the fascial continuum that extends to neck and arm.

### 2.5 Statistical methods and analysis

Qualitative variables are reported as absolute and relative frequencies, whereas quantitative variables are reported as mean and standard deviations. In order to assess the differences before therapy and after 9 months of follow up in cervical pain according to VAS (vas_Cerv) and in brachial pain according to VAS (vas_Br) the paired t-test was used. Additionally, a Linear Mixed Effects Models Trees (LMEMT from now on) proposed by Fokkema et al. (33) was used in order to search for different groups of patients where the evolution of Vas_Cerv and Vas_Br was different. This approach was used in two different settings: in the first one, variables Gender, Age, Symptoms Onset (SO), and Side (L/R affected upper limb) were used to find profiles of patients with different evolution of VAS scores. In the second approach variables Satisfaction, Pain, Range of motion (ROM) of cervical region (ROM_Cerv) and upper limb's ROM (ROM_Brach) were used in order to find profiles of perception associated to evolution of VAS scores. *Post hoc* analysis of VAS scores between profiles (evolution and perception) groups were done by means of ANOVA test. Finally, in order to assess the correlation between the cervical pain in VAS (vas_Cerv) and brachial pain in VAS (vas_Br) scores, a linear mixed effects model was fitted between the two scores and derived the generalized R2 index for these models (34) as a surrogate for correlation. All statistical methods were done in software R version 4.0.2.

## 3 Results

**Figure 2:**
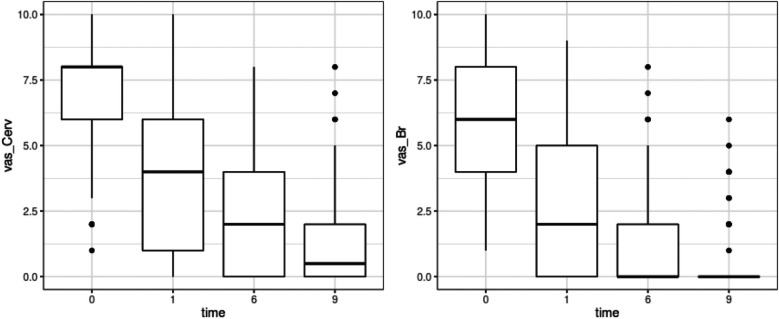
Results for endpoints of Vas_Cerv and Vas_Br evolution from baseline (month 0) pre-intervention and across all over the 9-months follow up period (time: 1, 6, 9 months).

The two primary endpoints were (i) changes in the cervical VAS pain and (ii) changes in the brachial VAS pain. The initial cervical pain VAS (vas_Cerv) score was high [Med 8 (Q1–Q3 = 6/8); M 7.148 (SD 2.147)]. After the Atlasprofilax therapy, there was a significant and continuous reduction in cervical pain VAS score throughout the study: 1 month [Med 4 (Q1–Q3 = 1/6); M 3.685, SD 2.858], 6 months [Med 2 (Q1–Q3 = 0/4); M 2.198, SD 1.996], and 9 months [Med 0.5 (Q1–Q3 = 0/2); M 1.475, SD 1.04]. The VAS difference or reduction in cervical pain after 9 months of therapy was significant, with a median of −6 (Q1–Q3 = −7/−4) and a mean of −5.673 (SD 2.308), *p* < 0.0001 (see Figure 2).

Brachial pain (vas_Br) had a median score of 6 (Q1–Q3 = 4/8) and a mean of 6.16 (SD 2.314) pre-intervention (Figure 2). A significant decrease in brachial pain was observed throughout the study, with progressive reductions in each subsequent evaluation: 1 month [Med 2 (Q1–Q3 = 0/5); M 2.772; SD 2.65]; 6 months [Med 0 (Q1–Q3 = 0/2); M 1.179, SD 1.794]; 9 month [Med 0 (Q1–Q3 = 0/2); M 0.333, SD 1.794]. The VAS difference at the 9-month follow-up had a median of −6 (Q1–Q3 = −8/−4) and a mean of −5.827 (SD 2.353), *p* < 0.0001 (Figure 2).

Overall cervical VAS pain score (vas_Cerv) diminished 5.6 units (SD 2.308, *p*-value <0.0001) and brachial VAS pain score (vas_Br) diminished 5.827 units (SD 2.353, *p*-value <0.0001) units at the 9 month follow up period (Figure 2).

**Figure 3:**
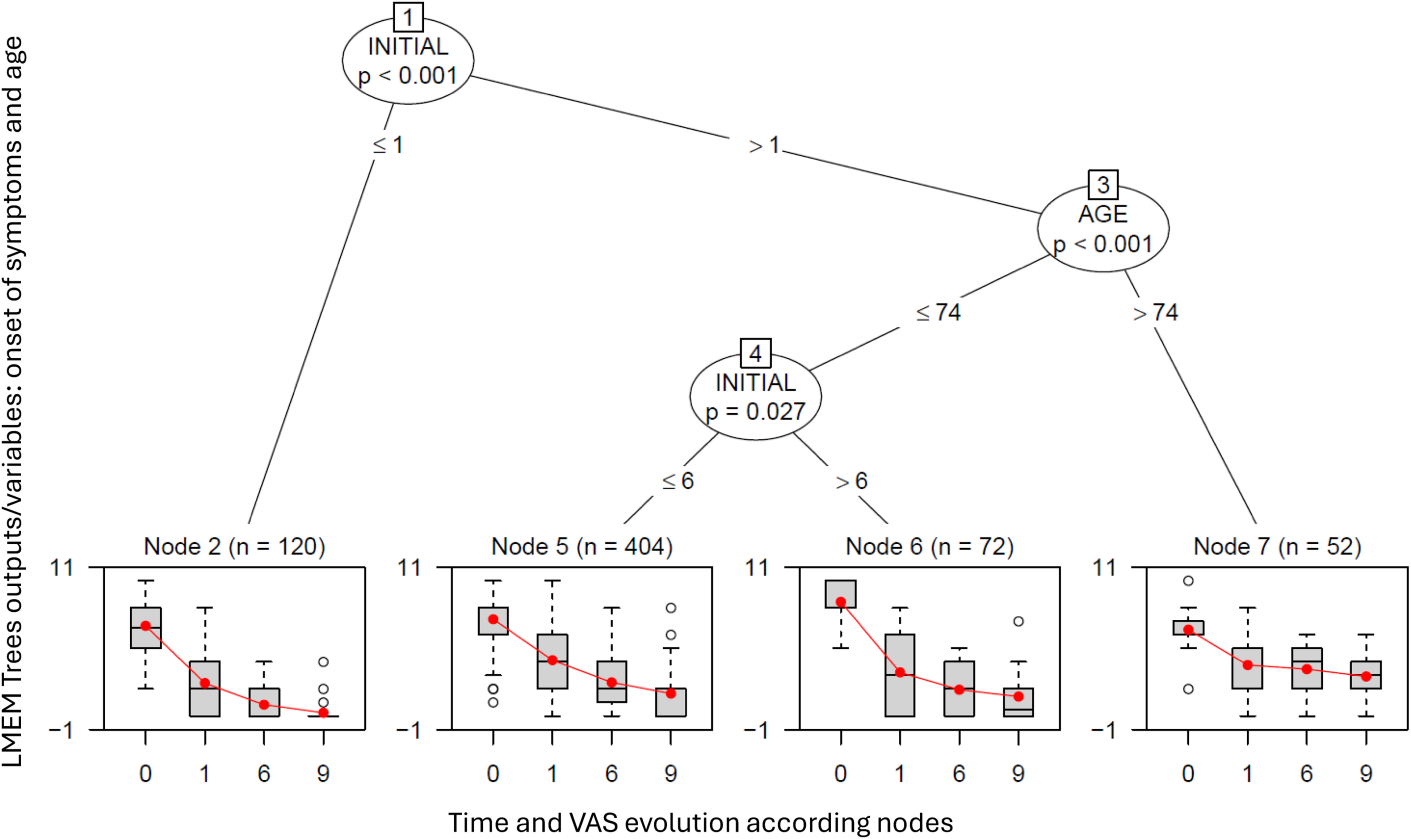
LMEMT for cervical VAS (vas_Cerv) evolution profiles. This tree was grown using variables Gender, Age, SO (Symptoms onset) and Side (L/R arm). Variables SO (Symptoms onset) and AGE differentiated 4 groups of patients with varying outcomes of vas_Cerv after therapy. Group 2 (Node 2, *n* = 30) had patients with 1 year or less of SO, with an initial vas_Cerv of 6.667 (SD 2.44) and a 9-month post-therapy score of 0.233 (SD 0.817), with a net reduction of −6.433 (SD 2.269), *p* < 0.0001. Group 5 (Node 5, *n* = 101) had patients with SO values >1 year but ≤6 and age ≤74, where vas_Cerv decreased from 7.158 (SD 2.106) pre-intervention to 1.663 (SD 1.785) at 9 months, with net reduction of −5.495 (SD 2.115), *p* < 0.0001. Group 6 (Node 6, *n* = 18) was characterized by patients with SO values >6 years and age ≤74, with an initial vas_Cerv of 8.444 (SD 1.504) decreasing to 1.444 (SD 1.947) at 9 months, leading to a net reduction of 7 (SD 2.59), *p* < 0.0001. Finally, Group 4 (Node 7, *n* = 13) had patients with SO values >1 year and age >74, with an initial vas_Cerv of 6.385 (SD 1.895) decreasing to 2.923 (SD 1.801) at 9 months, resulting in a net reduction of −3.462 (SD 1.613), *p* < 0.0001.

When using the LMEMT method to assess factors influencing the effectiveness of treatment in the primary endpoint of reducing cervical VAS, we observed the following grouping (see Figure 3).

Variables SO (Symptoms onset) and AGE differentiated 4 groups of patients with varying outcomes of vas_Cerv after therapy. Group 2 (Node 2, *n* = 30) had patients with 1 year or less of SO, with an initial vas_Cerv of 6.667 (SD 2.44) and a 9-month post-therapy score of 0.233 (SD 0.817), with a net reduction of −6.433 (SD 2.269), *p* < 0.0001. Group 5 (Node 5, *n* = 101) had patients with SO values >1 year but ≤6 and age ≤74, where vas_Cerv decreased from 7.158 (SD 2.106) pre-intervention to 1.663 (SD 1.785) at 9 months, with net reduction of −5.495 (SD 2.115), *p* < 0.0001. Group 6 (Node 6, *n* = 18) was characterized by patients with SO values >6 years and age ≤74, with an initial vas_Cerv of 8.444 (SD 1.504) decreasing to 1.444 (SD 1.947) at 9 months, leading to a net reduction of 7 (SD 2.59), *p* < 0.0001. Finally, Group 4 (Node 7, *n* = 13) had patients with SO values >1 year and age >74, with an initial vas_Cerv of 6.385 (SD 1.895) decreasing to 2.923 (SD 1.801) at 9 months, resulting in a net reduction of −3.462 (SD 1.613), *p* < 0.0001.

See Table 1 for a complete characterization of the cervical VAS evolution for each time period in each group of the tree.

**Table 1:** Description of vas_Cerv evolution for each one of the groups of the LMEMT evolution profiles (baseline, Cervical Vas at month 0 pre-intervention; C1M, Cervical Vas at 1 month; C6M, Cervical Vas at 6 months; CM9; Cervical Vas at 9 months; VAS_dif_C, decrease in vas_Cerv between baseline prior to intervention and at month 9).

vas_Cerv	Node 2	Node 5	Node 6	Node 7	*p*-value
Baseline	6.667 (2.44)	7.158 (2.106)	8.444 (1.504)	6.385 (1.895)	0.01412472
C1M	2.433 (2.528)	4.129 (2.894)	3.222 (2.922)	3.769 (2.522)	0.03401817
C6M	0.833 (1.392)	2.485 (1.983)	1.944 (1.862)	3.462 (1.984)	3.7218 × 10^−5^
C9M	0.233 (0.817)	1.663 (1.785)	1.444 (1.947)	2.923 (1.801)	3.0366 × 10^−6^
VAS_dif_C	−6.433 (2.269)	−5.495 (2.115)	−7 (2.59)	−3.462 (1.613)	7.3452 × 10^−5^

Reported values correspond to mean and standard deviations.

As can be seen in Table 1 and Figure 2, there were marked and significant differences in terms of the reduction of cervical VAS at month nine when compared to Cervical VAS at baseline prior to the intervention. All the reductions observed in all LMEMT profiled groups or nodes where statistically significant. Taking into account all this, it can be stated that less time of symptoms onset (SO) seems to be associated with greater reduction of vas_Cerv. An older age and a longer duration of symptom onset seem to be associated with a lesser reduction of vas_Cerv which, however, was still statistically significant in all LMEMT nodes. Gender and affected upper limb side (L/R) had no impact on LMEMT analysis outcomes.

When using the LMEMT method to assess for factors influencing effectiveness of treatment, in terms of reduction of Vas_Br, the LMEMT model did not find any significant grouping of patients leading to different evolution profiles.

The previous results in terms of vas_Br and vas_Cerv revealed very different patterns at least in terms of factors determining different profiles of VAS evolution. For the case of vas_Cerv, variables SO and Age produced 4 different evolution profiles, while for vas_Br LMEMT did not find any groups for which the treatment produced different evolution profiles.

**Figure 4:**
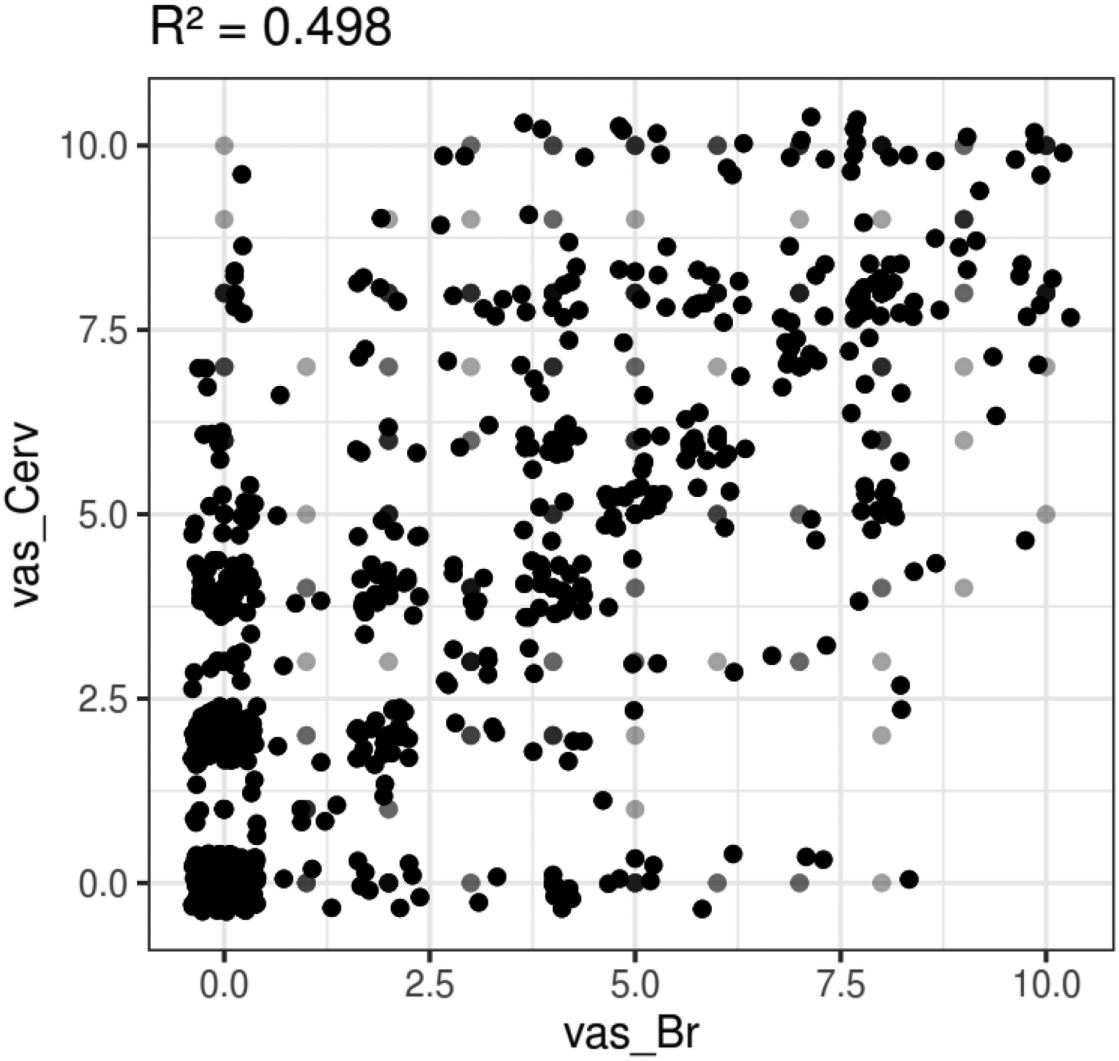
Degree of association between the vas_Cerv and the vas_Br according to a surrogate of measure of correlation with R2 for linear mixed models: scatterplot for vas_Cerv vs. vas_Br. Black dots indicate the presence of many values together, dark gray dots indicate the frequency of several values but less than black dots, light gray dots indicate a single value.

Taking this into account we assessed the degree of association between the vas_Cerv and the vas_Br. Figure 4 presents a dispersion diagram of the two VAS measures.

The R2 for linear mixed models was calculated as a measure of correlation, obtaining a value of 0.498, indicating a moderate positive correlation between neck pain and arm pain. However, it is important to note that correlation does not imply causation, and there could be multiple explanations for this correlation. One possible explanation is that neck pain and arm pain are both correlated to a common underlying condition, such as abnormalities in the suboccipital myofascia extending along the fascial continuum to the arm. Another possible explanation is that lifestyle or repetitive strain injury may also correlate with pain in the neck and arm. The presence of degenerative processes such as disc osteophytes found in 86 patients, cervical herniated disc (*n* = 9), and subjects presenting bad posture (*n* = 67), trauma, and other musculoskeletal disorders could also explain partially the R2 value of 0.498.

The outcomes for the secondary endpoints, which included (iii) changes in neck range of motion and (iv) changes in range of motion of the affected upper limb, are presented in Table 2.

**Table 2:** Range of motion of the cervical spine before and after application of the AtlasPROfilax method by using a goniometer (measurements in degrees).

Neck ROMs	Rotation		Inclination		Flexion		Extension	
	Before	After	Before	After	Before	After	Before	After
Average	33.0	60.4	25.1	41.8	56.9	75.5	39.5	62.9
SD	6.5	2.1	6.3	1.4	3.0	3.4	4.7	3.0
Upper limb ROMs	Flexion		Extension		Adduction		Abduction	
	Before	After	Before	After	Before	After	Before	After
Average	167.3	178.4	37.4	43.6	31.2	40.7	164.1	177.8
Sd	3.8	1.9	2.5	1.4	1.5	1.6	3.8	2.6

Range of motion of the upper limb before and after application of the AtlasPROfilax method by using a goniometer (measurements in degrees). Both measured at baseline (month 0) and at final follow-up (month 9).

The variation between the ROMs of the cervical spine at baseline and after 9 months resulted in an increase of 45.35% for rotation, 39.95% for inclination, 24.64% for flexion, and 37.20% for extension. The greatest variation in range of motion of the cervical spine occurred in rotation. The variation in terms of ROM amelioration for the affected upper limb after 9 months was 6.22% for flexion, 14.22% extension, 23.34% adduction, and 7.71% abduction. The greatest variation in range of motion of the upper limb occurred in adduction.

Regarding the last secondary endpoint, (v) patient final satisfaction, which was simply assessed by “Satisfied” or “Non satisfied”. Satisfaction of the treatment was informed by 141 (87.04%) patients, reporting improvement in their daily activities and pain notable reduction or disappearance. In the cases with total pain improvement (both cervical and brachial VAS = 0), the symptoms disappeared in an average of 180 days, where symptoms such as paresis were usually the first to disappear. Interestingly, there was no worsening in the VAS pain at the 9th month follow up among the non-satisfied patients. No side-effects related to the Atlasprofilax Method were reported during this study.

### 3.2 Limitations and future research

In single-arm studies, it is often challenging to distinguish the effects of the intervention from those of the placebo, the regression to mean and the natural disease course. While some results may be attributed to the intervention's efficacy, the placebo effect and spontaneous symptom improvement must also be considered. Therefore, single-arm studies are typically employed when the natural history of the condition is well-understood, when placebo effects are negligible or non-existent, or when participants are unlikely to improve spontaneously. In this study, the selected subjects had been suffering from chronic cervicobrachialgia for at least one year, with a mean onset of symptoms of four years. Patients with acute pain crisis or worsened symptoms were excluded to prevent possible contamination of the study's objectives by spontaneous improvement. Since patients experienced chronic, daily, and recurrent symptoms, the likelihood of bias and regression to the mean or spontaneous improvement by natural evolution of the condition was reduced.

According to a systematic review with a meta-analysis of 60 randomized controlled trials (RCTs), the placebo effect and responses accounted for 38% of neck pain score improvement in the active groups (35). This meta-analysis by Tengyue et al. found that the mean improvement in the visual analog scale (VAS) pain score after placebo treatment was 15.65% [mean difference (MD) = −15.65, 95% CI (−19.19, −12.12); *p* < .05], defined as the placebo response. In the active groups, the mean improvement in the neck pain score was 25.91% [MD = −25.91, 95% CI (−29.15, −22.68); *p* < .05], and in the no-treatment groups, it was 5.80% [MD = −5.80, 95% CI (13.28, 1.69); *p* = .13].

Although our study did not have a placebo control group, the active responder rate (RR) of ≥50% reduction in cervical VAS pain between baseline and 9-month follow-up achieved a value of 91.97% (*n* = 149/162), with a mean decrease in cervical pain of 85.20% [95% CI (88.08: 82.31)] within the ≥50% RR (*n* = 138/162). When comparing this active responder rate value against the values found by Tengyue et al. of 15.65% (placebo), 25.91% (active groups), and 5.80% (no treatment), and against the 38% pain score improvement accounted for by the placebo effect in the active groups, statistical significance was observed in favor of our study results (*P* < 0.0001).

The statistical significance was preserved when summing up a value of 63.91%, which included a 38% reduction in cervical VAS pain due to the placebo effect in the active groups and a 25.91% reduction in cervical pain observed in active groups in Tengyue et al.'s meta-analysis of 60 RCTs.

**Figure 5:**
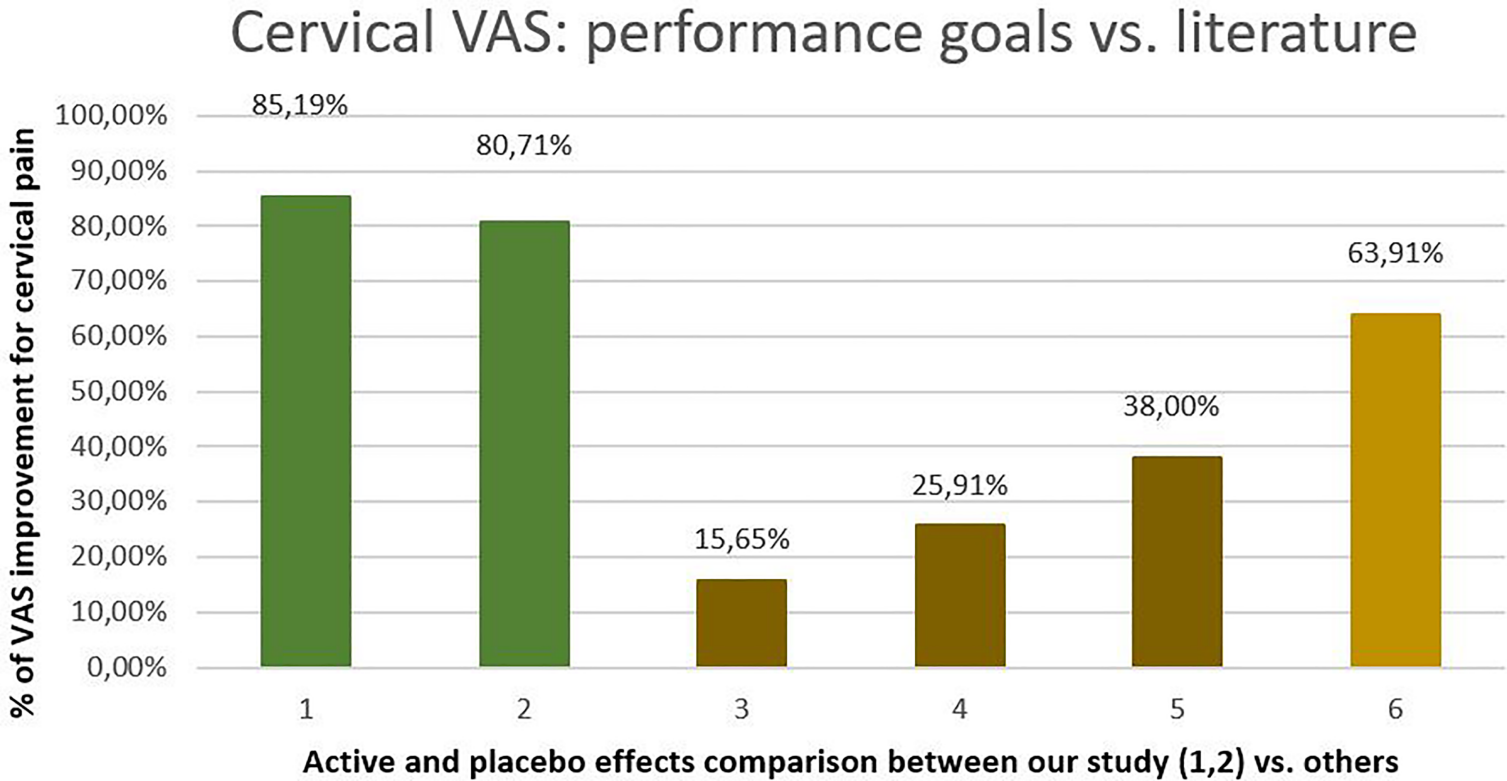
Comparation of our performance goals: (1) this study: overall mean cervical Vas pain reduction among RR ≥ 50% group (*n* = 149/162) and (2) this study: overall performance cervical VAS pain reduction (*n* = 162/162) (3) tengyue et al. cervical VAS after placebo, (4) Tengyue et al. cervical VAS reduction in active groups, (5) Tengyue et al. 38% for the pain score improvement in the active groups accounted for placebo effect, (6) Sum of the placebo effect accounted in the active groups (38%) according to Tengyue et al. plus the effect in the active group (25.91%).

In our study, 91.97% of patients (149/162) experienced at least a 50% reduction in cervical pain VAS (≥50% RR cervical VAS pain reduction), with mean reduction of 85.19% [95% CI (88.08: 82.31)] within the RR ≥ 50 group (See Table 3 above). Segner et al. validated a methodology for using external control groups for single-arm trials. In these cases, an external control group can deliver appropriate context for a single-arm trial, enabling causal inferences on treatment benefit (36). Thus, we established several performance goals to compare them against the values found in the Tengyue et al. that included the results of 60 RCTs (see Table 4 and Figure 5). Since our overall performance in cervical pain reduction was 80.71%, 95% CI [84.29: 77.13] (*n* = 162/162), and the mean VAS neck pain reduction among ≥50% RR group was 91.97% [95% CI (95.55: 88.39), *n* = 149/162], the null hypothesis was rejected in all three cases, as the lower limits of the 95% CI were greater than 38% and 63.91%, achieving the performance goals (see Table 4 and Figure 5).

**Table 3:** Cervical VAS pain reduction in this study.

(A) Cervical VAS ≥ 50% RR for pain reduction among in this study				
	Reduction < 50%	Reduction ≥ 50%	CI 95%	*P*-value
*N* = 162	13 (8.03%)	149 (91.97%)	91.97% (95.55: 88.39)	<0.0001
(B) Overall cervical VAS pain 100% reduction in this study				
	Reduction <100%	Reduction = 100%	CI 95%	*P*-value
*N* = 162	81 (50%)	81 (50%)	–	–
(C) Mean cervical VAS pain reduction among RR ≥ 50% group at month 9 (*n* = 149/162)				
	Mean (in RR ≥ 50% group)		CI 95%	*P*-value
*N* = 149	85.19%		85.19% (88.08: 82.31)	<0.0001
(D) Overall performance of cervical VAS pain reduction in this study at month 9 (*n* = 162)				
	Mean (overall)		CI 95%	*P*-value
*N* = 162	80.71%		80.71% (84.29: 77.13)	<0.0001

**Table 4:** Description of the performance goals 1–6: cervical VAS pain mean improvement in this study (in overall and RR ≥ 50% groups) and cervical VAS pain mean improvement found in Tengyue et al. (placebo, active, both combined, and all summed groups).

Performance goals. (study/description)	Cervical VAS pain mean improvement
1. This study: Overall mean cervical VAS pain reduction among RR ≥ 50% group (*n* = 149/162; 91.97%)	85.19% [95% CI (88.08: 82.31)]
2. This study: Overall performance in mean cervical VAS pain reduction (*n* = 162/162)	80.71% [95% CI (84.29: 77.13)]
3. Tengyue et al.: Mean cervical VAS pain reduction after placebo	15.65% [95% CI (19.19: 12.12)]
4. Tengyue et al.: Mean cervical VAS pain reduction effect in active groups	25.91% [95% CI (29.15: 22.68)]
5. Tengyue et al. for the pain score improvement in the active groups accounted for placebo effect	38.00%
6. Sum of the placebo effect (38%) in the active group plus active group placebo effect (25.91%) according to Tengyue et al. in 60 RCTs.	63.91%

Although a direct comparison cannot be made, our preliminary findings suggest that the Atlasprofilax effectiveness is significantly superior to the placebo effect accounted for the active groups and/or when combining both, placebo effect plus placebo effect accounted for the active groups, observed in 60 RCTs for neck pain. It is worth noting that the intervention was a single session lasting an average of 8 min, administered immediately after baseline measurements. Due to the absence of any other treatments during the 9-month follow-up period, the statistically significant and sustained improvement in cervical and brachial VAS total pain reduction [80.71%, 95% CI (84.29:77.13) and 95.28%, 95% CI (97.52:93.04), respectively] makes it unlikely that significant continuous placebo effect could account for these outcomes. This further supports the efficacy of the intervention. Nevertheless, these findings are preliminary and require validation through a double-blind study with three groups: intervention group, placebo control group, and no intervention group.

## 4 Discussion

The AtlasPROfilax intervention was administered to 162 patients resulting in a significant long-lasting reduction of cervicobrachialgia symptoms. At final 9-month follow-up, Vas pain was reduced with values of 80.71% and 95.28% for the patients with cervical and brachial complaints, respectively. The intervention also improved the all ranges of motion of both the cervical spine and affected upper limb and led to a patient satisfaction rate of 87.04% (*n* = 141/162).

Current pharmacological and surgical approaches to upper extremity manifestations of chronic cervicobrachialgia have limited efficacy and are often met with difficulties. The myofascial system is interconnected with neural and vascular structures, indicating that upper limb issues may result from abnormalities, chronic tensions, or structural and metabolic changes in the upper cervical region. The Atlasprofilax Method, which involves a device-mediated, non-invasive approach through controlled vibropressure intervention of suboccipital myofascia, appears to be with promising results that may complement other pre-surgical, conservative, pharmacological, or non-pharmacological therapeutic approaches.

Cervicobrachial pain is a common ailment, but only about 20% of cases are believed to have a neurogenic origin (37). This raises the possibility that other factors, such as deformations of the deep cervical fascia, dysfunctions of the myodural bridge, and alterations of the suboccipital myofascia, may contribute to the development of this condition (16). Previous research has suggested that underestimated alterations in the suboccipital myofascia may be involved in various chronic benign pain conditions (32). While the literature is not conclusive, it appears that in roughly 60% of cases, neck pain precedes arm pain, while in 30% of cases, arm pain is the initial symptom. The remaining 10% of patients experience both neck and arm pain at the same time.

In our study, we observed statistically significant reductions in both cervical and brachial pain. However, we noted that brachial pain tended to improve more rapidly and significantly than cervical pain (see Figure 2). We suggest that this observation may be explained by the distal source of upper limb pain caused by alterations originating in the suboccipital myofascia. We hypothesize that undetected structural and metabolic alterations of the suboccipital myofascia, deep cervical fascia, and neck muscles, especially the suboccipital muscles, may contribute to stress and pain along the neck-arm myofascial continuum (8, 16).

Therefore, it seems that symptoms of cervicobrachial pain may appear first in the cervical region and later extend to the upper limb according to the principles of fascintegrity (38), biotensegrity and myofascial chains (39). This is supported by the fact that cervical pain was significantly higher than brachial pain at baseline, and the R2 correlation coefficient of 49% suggests a suboccipital origin of cervicobrachialgia symptoms in the neck-arm myofascial continuum in at least half of the patients. About 53% of patients presented degenerative processes, which could interfere with the R2 correlation coefficient as an added co-factor for cervicobrachialgia but could also paradoxically explain that even in the presence of such cervical degenerative conditions, the neck-arm myofascial continuum still responded well to the suboccipital myofascial release stimulation. This is reflected in the significant reduction of cervical and brachial VAS scores at month 9. None of the 162 patients received any other co-adjuvant therapies for cervicobraquialgia symptoms during the study period.

Restoring or improving the upper cervical myofascial stress and imbalance through a mechanotransductive stimulus on the suboccipital myofascia may result in the disappearance of distal symptoms first, as observed in Figure 2. It should be noted that the intervention was performed only once on the suboccipital myofascia, and no intervention was carried out on the affected upper limb.

According to Janda, functional dysfunction arises due to chronic muscle imbalance (40). The growth and remodeling of load-bearing biological soft tissues have been well-described by Cyron & Humphrey (41) explaining some biomechanical and biochemical mechanisms of adaptation in the myofascia. This means that the myofascia may respond in the mid-to-long term with a defensive or adaptation compensation mechanism, ultimately delaying or differing the onset of distal impairments and pain to structures such as the affected upper limb. However, the adaptation mechanisms of fascia involving its biomechanics and metabolism may challenge its adaptation ability through hysteresis and fascial adaptation's capabilities in ECM and cellular homeostatic control.

Therefore, if suboccipital alterations persist as the main problem's origin, those adaptations could ultimately result in fascial creep. As a result, fibroblastosis, fascial and fiber entanglement, alteration of mechanoreceptors and nociceptors, and even affectation of the microvacuolar fascia may arise, leading to vessel and nerve affectation. These suboccipital alterations could be improved by the Atlasprofilax intervention explaining the observed results.

Further studies are recommended to evaluate this innovative intervention. It is also advisable to study the possible relationships and connections between biomechanical and metabolic alterations of the suboccipital region, especially suboccipital myofascia, and their impact through the fascial continuum in cervicobrachialgia. The presence of commonly unnoticed or undetected suboccipital myofascia affectations may partially underlie the symptomatology and etiology of cervicobrachialgia. Further research is needed to confirm these findings and explore the potential benefits of treatments targeting the suboccipital myofascia. To conclude, AtlasProfilax is nowdays as an non pharmacologically alternative intervention when it comes to pain control and activities of daily living, in patients with cervicobrachialgia.

## Data Availability

The data presented in the study are deposited in the Mendeley Data, V1, repository, accession number doi: 10.17632/7gzd935fvy.1 s.
